# Inhibition of ADAM10 ameliorates doxorubicin-induced cardiac remodeling by suppressing N-cadherin cleavage

**DOI:** 10.1515/biol-2021-0081

**Published:** 2021-08-27

**Authors:** Xiaoou Li, Feng Pan, Bing He, Chengzhi Fang

**Affiliations:** Department of Neonatology, Renmin Hospital, Wuhan University, Wuhan, Hubei 430060, People’s Republic of China; Department of Orthopedics, Renmin Hospital, Wuhan University, Wuhan, Hubei 430060, People’s Republic of China; Department of Pediatrics, Renmin Hospital, Wuhan University, Wuhan, Hubei 430060, People’s Republic of China

**Keywords:** ADAM10, N-cadherin, cell adhesion, shedding, DCM, cardiac remodeling

## Abstract

The present research was designed to examine the effects of disintegrin metalloproteinases 10 (ADAM10) on the doxorubicin (DOX)-induced dilated cardiomyopathy (DCM) and the mechanisms involved, with a focus on ADAM10-dependent cleavage of N-cadherin. The present study constructed recombinant lentiviral vectors expressing short hairpin RNA (shRNA) targeting the ADAM10 gene. H9C2 cells were treated with the recombinant lentivirus or GI254023 (an ADAM10 inhibitor). The expression level of N-cadherin and its C-terminal fragment1 (CTF1) was tested by western blotting and flow cytometry. The adhesion ability was analyzed using a plate adhesion model. Cardiac function and morphology were assessed in control and lentivirus-transfected rats with or without DOX treatment. The inhibition of ADAM10 activity significantly increased the expression of full-length N-cadherin on the cellular surface and reduced CTF1 generation *in vivo* and *in vitro*. The adhesion ability was also increased in ADAM10-knockdown H9C2 cells. Furthermore, DOX-induced myocardial dysfunction was ameliorated in rats transfected with ADAM10-shRNA lentivirus. These findings demonstrated that ADAM10 specifically cleaves N-cadherin in cardiomyocytes. ADAM10-induced N-cadherin cleavage results in changes in the adhesive behavior of cells. Therefore, ADAM10 may serve as a therapeutic target to reverse cardiac remodeling in DCM.

## 1 Introduction

Dilated cardiomyopathy (DCM) is a severe cardiac disorder with structural and functional myocardial abnormalities [1], which leads to high morbidity and mortality as a result of complications，including heart failure [2]. The clinical progression of congestive heart failure is largely determined by the dynamic and continuous progression of cardiac remodeling [3]. Recent studies have suggested that cardiac remodeling may be a global myocardial process involving left ventricular contractile function as well as mitral regurgitation, left ventricle diastolic function, and the right ventricle [4,5]. The main aim in the treatment of DCM is therapy-induced reverse remodeling. Therefore, reverse remodeling of DCM-affected hearts is one of the most promising research targets.

The coupling of neighboring myocytes is mediated by the intercalated disc (ID) through intercellular signaling, which controls the cardiomyocyte remodeling and function [6]. It is well-known that the DCM exhibits a highly irregular structure associated with alterations of the ID components, especially the abnormal expression of proteins comprising the adherent junctions (AJs) [7]. N-cadherin is an integral component of AJs, which functions to mechanically and electrically couple adjacent cardiomyocytes. Mice with altered expression of N-cadherin showed a DCM phenotype, probably due to the malfunction of the ID [8]. Similarly, mice with the specific knockout of N-cadherin in cardiomyocytes exhibited disrupted assembly of ID, impaired cardiac function, and a loss in muscular tension and died within two months of transgene expression [9].

The ectodomain of N-cadherin is essential for the regulation of cell adhesion and cell migration [10]. Previous studies have suggested that disintegrin metalloproteinases 10 (ADAM10), a member of the ADAM family, is involved in the ectodomain cleavage of N-cadherin in fibroblasts and neurons. In addition, intercellular adhesion is upregulated in ADAM10-deficient fibroblasts and neurons [11,12]. Nonetheless, whether ADAM10 is responsible for releasing the extracellular domain of N-cadherin in cardiomyocytes remains unresolved.

ADAM10 has been studied in the context of the proteolysis of various substrates, including cytokines, growth factors, and adhesion molecules, and plays important roles in fertilization, neurogenesis, and angiogenesis [13,14]. More recent studies indicated that ADAM10 is markedly elevated in human DCM, and ADAM10 expression is reduced after reverse remodeling resulting from mechanical unloading [15,16]. However, insights into the role of ADAM10 in cardiac remodeling are still at the preliminary stage.

The present study focused on the effects of ADAM10 on N-cadherin ectodomain cleavage in cardiomyocytes. Furthermore, the study examined how ADAM10 affected the intercellular adhesion abilities and regulated the cardiac remodeling and demonstrated that inhibition of ADAM10 has therapeutic benefits against doxorubicin (DOX)-induced cardiac dysfunction and remodeling.

## 2 Materials and methods

### 2.1 Construction and identification of ADAM10-short hairpin RNA (shRNA) lentiviral vectors

The shRNA sequence that was applied to target ADAM10 was used as previously described [11]. Synthetic oligonucleotide sequences were constructed and annealed to create double-stranded DNA using the following sequences: Forward, 5′-AACTCAGTGTGCATTCAAGTCAATTCAAGAGATTGACTTGAATGCACACTGTTTTTTC-3′ and reverse, 3′-TCGAGAAAAAACAGTGTGCATTCAAGTCAATCTCTTGAATTGACTTGAATGCACACTGAGTT-5′. The target gene was inserted into the *Hpa* I- and *Xho* I-cleaved LentiLox3.7 vectors via homologous recombination using the T4 DNA ligase enzyme. Competent DH5α cells were prepared with calcium chloride and subsequently transformed. Positive clones were selected by PCR analysis using Taq enzyme (cat. no. DR010s; Takara Biotechnology Co., Ltd.). Recombinant positive clones were sent to Invitrogen (Thermo Fisher Scientific, Inc.) for sequencing.

### 2.2 Virus packaging and titer assessment

A total of 1 × 10^6^ HEK-293 cells/mL were plated prior to transfection. Plasmid DNA (3 μg:1.5 μg of shuttle plasmid and packaging plasmid) and 6 μL of TurboFect transfection reagent were diluted in 200 μL of Opti-MEM. Subsequently, the mixture was incubated overnight at 37°C. The media was changed after 16 h. Conditioned medium was harvested after an additional 48 h, purified by centrifugation, filtered through a polyvinylidene fluoride (PVDF) filter (0.45 μm), and stored at −80°C.

HEK-293 cells were plated into six-well plates (2 × 10^5^ cells/well) 24 h prior to transfection. Virus stocks were serially (10^−2^ to 10^−6^) diluted into 100 μL of complete medium containing polybrene. Serial dilutions were added to three wells for each density with the control media. The media were changed 24 h later. Following 48 h in culture, green fluorescent protein (GFP) expression in each well was observed under a fluorescence microscope (magnification ×200). The number of cells expressing GFP was counted and multiplied by the dilution ratio to determine the viral titer (transducing units [TU]/mL).

### 2.3 Treatment of H9C2 cells with ADAM10-shRNA lentiviral virus or ADAM10 inhibitor

H9C2 cells (Cell Bank, Chinese Academy of Sciences) were cultured in high glucose DMEM supplemented with 10% of fetal bovine serum. Next day, an appropriate amount of virus (MOI = 20) was diluted into the culture medium (containing 8 μg/mL of polybrene). Then, the recombinant viral supernatant and control viral supernatant were added. Subsequently, the old media were replaced with virus-containing media, and the cells were incubated overnight. On the following day, the virus-containing media were removed and replaced with fresh culture media. H9C2 cells were harvested 72 h after transfection, and the total RNA and protein were isolated. H9C2 cells were treated with hydroxamate-based inhibitor GI254023 (Bio-Techne) as described previously [17]. For FACS analysis and adhesion assays, the cells were harvested under cadherin-saving conditions.

### 2.4 Flow cytometry analysis

Cells were stained with fluorescence-labeled anti-N-cadherin antibody (cat. no. ab195185; 1:1,000 dilution; Abcam) in PBS for 30 min at 4°C, while cells in the control group were treated with PBS alone. Following washing with PBS twice, the cells were resuspended in PBS at 4°C and immediately subjected to flow cytometry analysis (BD FACSCalibur; BD Biosciences, Franklin Lakes, NJ, USA). The average fluorescence of 5 × 10^4^ cells was used to determine the expression levels of N-cadherin.

### 2.5 Cell adhesion assay

The 96-well plates were coated with fibronectin (10 μg/mL; 100 μL/well; Merck KGaA). Wells coated with 1% of BSA and 100 μg/mL of polylysine were used as a reference for minimum and maximum adhesion, respectively. Cell suspension (5 × 10^5^ cells/mL; 100 μL) from the ADAM10-shRNA transfection group, mock transfection group, or blank control group was added, followed by incubation for 3 h at 37°C. PBS was used to rinse off non-adherent cells. The cells were then fixed with paraformaldehyde and stained with 1% of methylene blue for 20 min. Subsequently, 100 μL of HCl (1 mol/L) was added, followed by incubation at 37°C for 40 min. The absorbance (*A*) at 600 nm was detected by using a microplate reader. The cell adhesion rate was calculated using the following formula:

({A}_{\text{test}\text{well}}-{A}_{\text{BSA}\text{well}})/({A}_{\text{polylysine}\text{well}}-{A}_{\text{BSA}\text{well}})\times 100 \% \text{.}]

### 2.6 Establishment of model and treatment

Adult male Sprague-Dawley rats (eight weeks old, purchased from the Experimental Animal Center of Wuhan University, Wuhan, China) were randomized into four groups: Normal control group (*n* = 25), DCM model group (*n* = 25), ADAM10-shRNA group (*n* = 25), and empty vector group (*n* = 25). Two or three rats were housed per cage under specific pathogen-free conditions (controlled temperature of 24 ± 3°C and humidity of 55 ± 15%) with a 12 h light/dark cycle and *ad libitum* access to standard food and tap water. The rats were anesthetized via sodium pentobarbital (30 mg/kg) prior to the ventricular injection. Animals were allowed a 3 day’s acclimatization period prior to the experimental protocol. The ADAM10-shRNA lentiviral vectors (1 × 10^9^ pfu/mL) were injected into the left ventricle cavity through the heart apex in the ADAM10-shRNA group prior to DOX injection, whereas rats from the vector group received equal volumes of empty vector [18]. After 3 days, 4 rats in the ADAM10-shRNA group were used to isolate primary myocardial cells in order to detect fluorescence. The other rats were treated with or without intraperitoneal (i.p.) injection of 2 mL/kg DOX (Merck KGaA) once a week. If rats showed signs of severe lethargy, such as not responding to cage handling or touch, ascites, increasing respiratory effort or respiratory distress, and substantial weight loss (20%), they would be euthanized. Six weeks later, rats were euthanized via pentobarbital overdose (i.p.: 100 mg/kg) injection. The surgery was performed under sodium pentobarbital (30 mg/kg) anesthesia. All efforts were made to minimize suffering.

**Ethical approval:** The research related to animal use has been complied with all the relevant national regulations and institutional policies for the care and use of animals. The protocol was performed in compliance with the guidelines approved by the Animal Care and Use Committee of the Wuhan University (no. WDRY2013-L016).

### 2.7 Detection of protein expression by western blotting

Total protein extracted from heart tissues post-DOX injection and H9C2 cells transfected with ADAM10-shRNA lentiviral vectors were fractionated by SDS-PAGE, then transferred to PVDF membranes (EMD Millipore). After blocking with BSA for 1 h, membranes were incubated with primary rabbit anti-human anti-ADAM10 (cat. no. ab124695; 1:1,000; Abcam) or anti-N-cadherin antibody (cat. no. ab18203; 1:1,000; Abcam), followed by incubation overnight at 4°C. The primary antibody for N-cadherin also recognizes antigenic epitopes of C-terminal fragment1 (CTF1); hence, the N-cadherin antibody was also used as the CTF1 antibody. Subsequently, membranes were washed three times with TBS-Tween 20 and incubated with a secondary HRP-labeled goat anti-rabbit antibody (cat. no. sc-2004; 1:1,000; Santa Cruz Biotechnology, Inc.) at room temperature for 1 h. The film was scanned and analyzed using a gel imaging processing system (GSD800 system; Beijing Sage Creation Science Co., Ltd.). The results were analyzed using Quantity One software v4.62 (Bio-Rad Laboratories, Inc.). GAPDH (cat. no. ab8245; 1:1,000; Abcam) was used as the internal control. The experiment was repeated three times.

### 2.8 Echocardiography

Following DOX injection, echocardiography was performed using an animal-specific instrument (Vevo707B; Visual Sonics Inc.). The four groups were anesthetized by isoflurane inhalation (2% for induction and 1.5% for maintenance), and M-mode images were recorded when the rats’ heart rates were maintained at 450–500 bpm. The left ventricular ejection fraction (LVEF), left ventricular end-diastolic dimension (LVEDD), and left ventricular fractional shortening (LVFS) were measured as previously described [19]. All measurements were averaged from five consecutive cardiac cycles and were performed by three experienced technicians who were unaware of the identities of animal groups.

### 2.9 Morphological and histological evaluation

After the rats were euthanized, the hearts were harvested post-DOX injection, fixed with 4% of paraformaldehyde, and embedded in paraffin. The sections (4 μm) were stained with hematoxylin-eosin (H&E). Stained tissues were observed under a fluorescence microscope (BX53; Olympus Corporation) and the images were captured for analysis; a total of ten fields of view were observed. For immunohistochemistry (IHC) analysis, paraffin-embedded sections were deparaffinized in xylene and rehydrated in graded ethanol. Heat mediated antigen retrieval was performed to expose antigen epitopes. The sections were incubated with N-cadherin antibody that was also used for western blotting analysis (1:150; at 4°C; overnight). Samples were further processed using a goat anti-rabbit IgG IHC kit (cat. no. PV-9001; OriGene Technologies, Inc.), and the sections were counterstained with hematoxylin.

### 2.10 Statistical analysis

All experiments were repeated at least three times. Analyses were performed with JMP 7 (SAS Institute. Inc.) and GraphPad Prism 7 (GraphPad Software. Inc.). Data were expressed as the mean values ± SEM, and analyzed for normality using the D’Agostino-Pearson Omnibus test and *F*-test for equality of variance. For comparisons of the two experimental conditions of normal data, statistical significance was determined by Student’s *t*-test or Welch’s *t*-tests; for comparisons of three or more experimental conditions, repeated-measures analysis of variance (ANOVA) was used, followed by a Tukey’s post hoc analysis. The Mann–Whitney *U* test was used for comparisons between two groups, and the Kruskal–Wallis test was used for multiple comparisons in non-normally distributed data. Wilcoxon’s signed-rank test was used for non-parametric paired data samples. *P* < 0.05 was considered to indicate a statistically significant difference.

## 3 Results

### 3.1 Construction of ADAM10-shRNA lentiviral vectors

**Figure 1:**
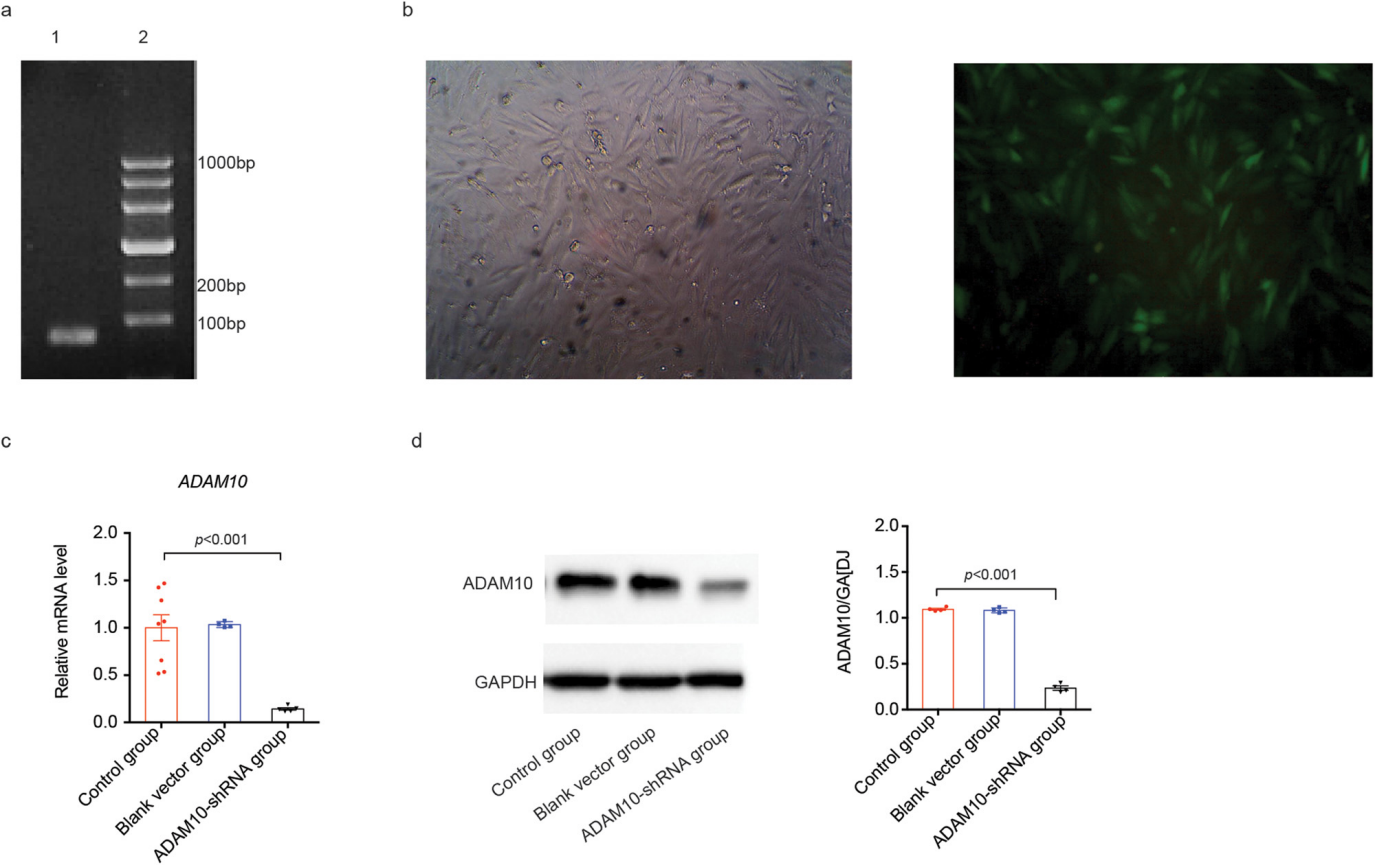
Construction of ADAM10-shRNA lentiviral vectors. (a) PCR products of LentiLox-ADAM10-shRNA-transfected cells. (b) Fluorescence images of H9C2 cells transduced with ADAM10-shRNA lentiviral vectors for 3 days; GFP was observed under light (left) or fluorescence (right) microscopy. Analysis of (c) ADAM10 mRNA levels and (d) protein expression in H9C2 cells transduced with or without ADAM10-shRNA lentiviral vectors for 3 days. *P-*values in (c and d) were determined using the Kruskal–Wallis test. All data are presented as the mean values ± SEM. Magnification ×200. ADAM10, disintegrin metalloproteinase 10; shRNA, short hairpin RNA; GFP, green fluorescent protein.

The LentiLox-ADAM10-shRNA was constructed successfully. Recombinant positive clones that have incorporated the target shRNA sequence were identified using PCR and sequencing; the shRNA insert is 70 bp (Figure 1a). Subsequently, HEK-293 cells were transfected with the lentiviral package system. The lentiviral titer was identified according to the GFP expression observed under an inverted microscope. The final viral titer of the recombinant lentivirus was 8 × 10^8^ TU/mL, indicating efficient transfection and successful virus packaging.

To assess the effects of ADAM10-shRNA, H9C2 cells were transfected with the LentiLox-ADAM10-shRNA using Lipofectamine. As shown in Figure 1b, at 72 h post-transfection, approximately 90% of the transfected H9C2 cells expressed GFP, demonstrating that the ADAM10-shRNA lentivirus showed a potent tropism for H9C2 cells. qPCR (Figure 1c) and western blotting (Figure 1d) displayed that the ADAM10 protein levels significantly decreased in transfected cells compared with the control group, indicating that ADAM10-shRNA effectively silenced *ADAM10* gene and inhibited its protein expression.

### 3.2 ADAM10-dependent N-cadherin cleavage in H9C2 cells

**Figure 2:**
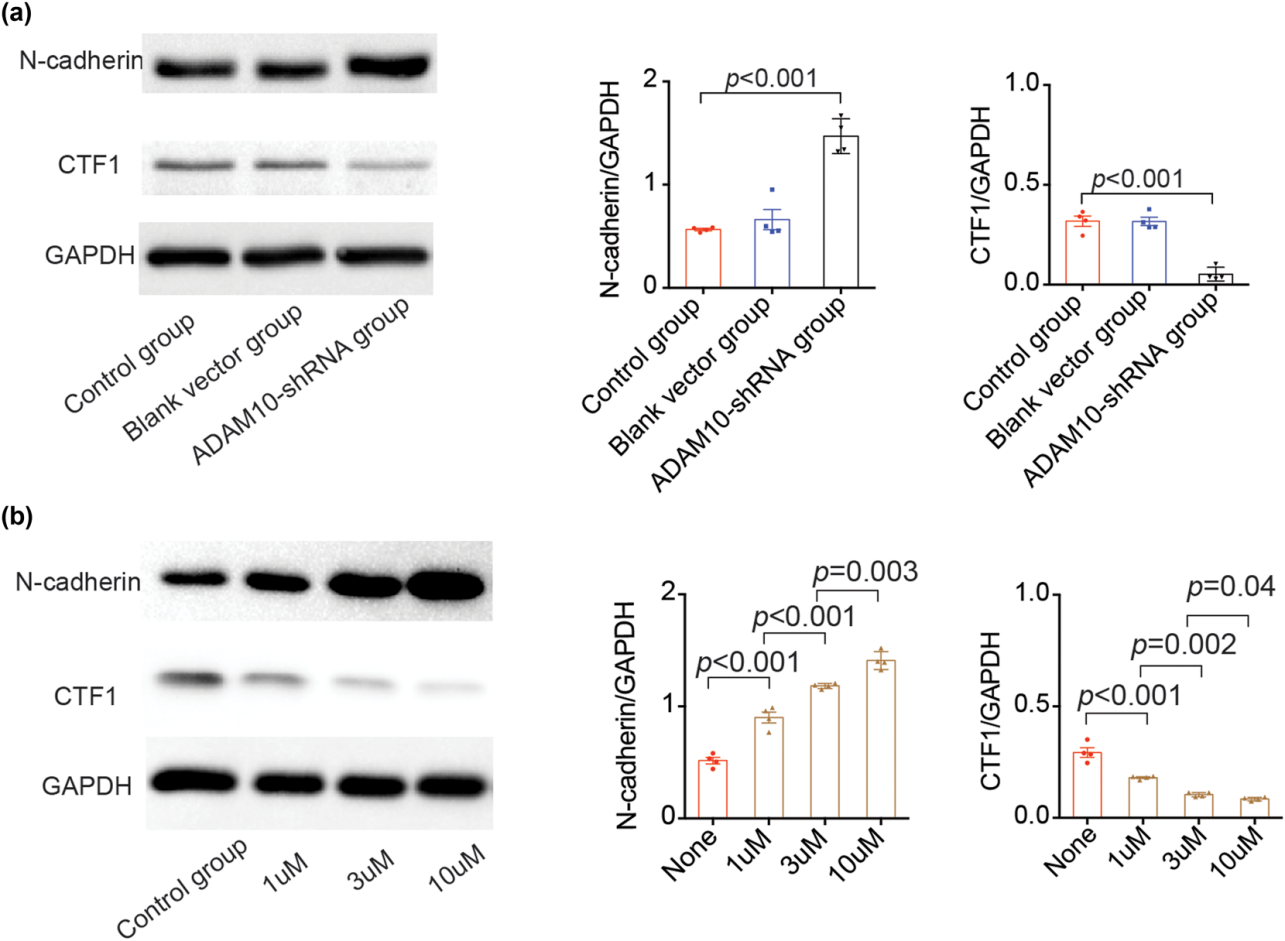
ADAM10-dependent N-cadherin cleavage in H9C2 cells. (a) Protein expression of N-cadherin and CTF1 in the H9C2 cells transfected with or without ADAM10-shRNA lentiviral vectors for 3 days. (b) Protein expression of N-cadherin and CTF1 in the H9C2 cells treated with various concentrations of ADAM10 inhibitor (GI254023X) or vehicle control (DMSO) for 4 h and analyzed by western blotting. *P-*values in (a and b) were determined using the Kruskal–Wallis test. All data are presented as the mean values ± SEM. ADAM10, disintegrin metalloproteinase 10; CTF1, C-terminal fragment 1; shRNA, short hairpin RNA.

ADAM10 is involved in the shedding of various membrane-bound proteins. N-cadherin is a transmembrane protein (135 kDa) cleaved by ADAM10 at its ectodomain (R714-I71), thereby generating CTF1 (40 kDa) in fibroblasts and neurons [20]. To assess the effects of ADAM10 in the ectodomain shedding of N-cadherin in cardiomyocytes, the H9C2 cells were transfected with ADAM10-shRNA lentiviruses. Western blotting showed that ADAM10 levels in the transfected group reduced to ∼20% compared with the control group, and CTF1 levels decreased to a similar extent, while N-cadherin protein expression was significantly increased (Figure 2a). Furthermore, the findings were confirmed via pharmacological means using GI254023X (an ADAM10 inhibitor) [17]. The inhibitor reduced the generation of CTF1 in H9C2 cells in a dose-dependent manner, demonstrating the essential role of ADAM10 in the hydrolysis process (Figure 2b).

**Figure A1:**
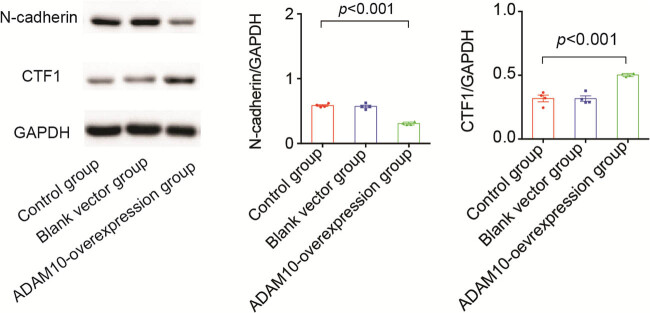
N-cadherin shedding was restored in H9C2 cells following transfection with wild-type ADAM10. Protein expression of N-cadherin and CTF1 in the H9C2 cells transfected with ADAM10 overexpression vectors for 3 days. *P-*values were determined using the Kruskal–Wallis test. All data are presented as the mean value ± SEM. ADAM10, disintegrin metalloproteinase 10; CTF1, C-terminal fragment1.

Finally, H9C2 cells completely regained N-cadherin shedding after being transfected with wild-type ADAM10. In addition, markedly increased levels of CTF1 were observed following ADAM10 overexpression (Figure A1). Taken together, the results demonstrated that ADAM10 is responsible for the N-cadherin ectodomain cleavage.

### 3.3 Cadherin-mediated intercellular adhesion is upregulated in ADAM10-knockout H9C2 cells

**Figure 3:**
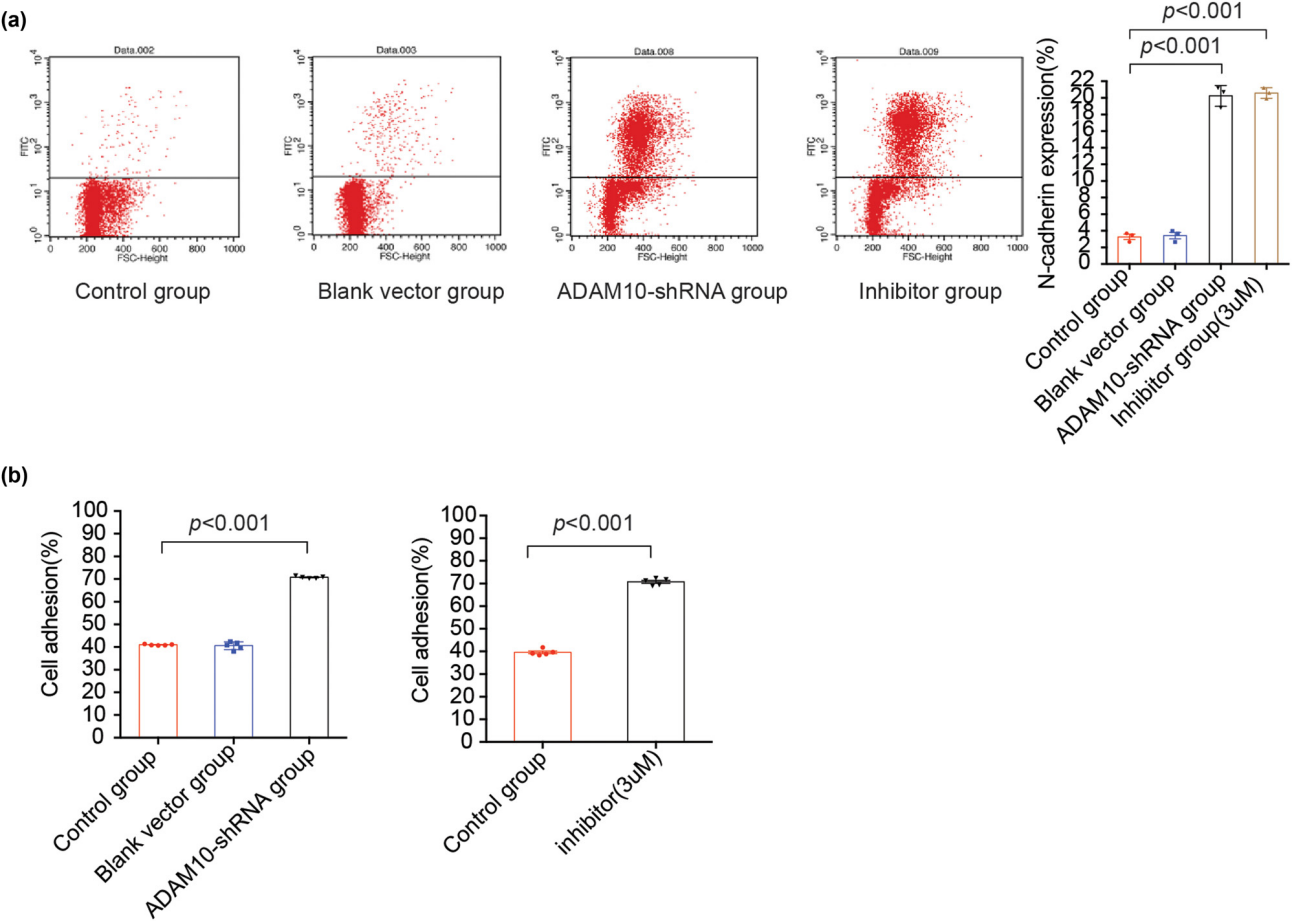
Cadherin-mediated intercellular adhesion is upregulated in ADAM10-knockout H9C2 cells. (a) Detection of N-cadherin on the surface of H9C2 cells transfected with ADAM10-shRNA or ADAM10 inhibitor by flow cytometry. (b) The adhesion ability of H9C2 cells transfected with ADAM10-shRNA (left) or ADAM10 inhibitor (right). *P-*values in a and b were determined using the Kruskal–Wallis test. All data are presented as the mean values ± SEM. ADAM10, disintegrin metalloproteinase 10; CTF1, C-terminal fragment 1; shRNA, short hairpin RNA.

The release of the extracellular domain of N-cadherin is functionally important in cell adhesion. The present research next evaluated the extent of ADAM10 effects on the surface expression of N-cadherin in H9C2 cells using flow cytometry analysis. Compared with that in the control group, full-length N-cadherin on the cell surface in the transfection group was significantly increased (Figure 3a). Similar results were also observed in the ADAM10 inhibitor group (Figure 3a). These results further indicated that inhibiting ADAM10 activity reduced the cleavage of the N-cadherin extracellular domain and increased the full-length N-cadherin form. In order to further understand this increase in cell adhesion, the effects of ADAM10-shRNA on the adhesion ability of H9C2 cells were investigated. The ADAM10-shRNA group showed a significantly higher adhesion than the control group (Figure 3b). The present study further confirmed these results in cells tested with ADAM10 inhibitor (Figure 3b). Taken together, these assays demonstrated that ADAM10-mediated N-cadherin ectodomain cleavage could influence the adhesion capability of myocardial cells.

### 3.4 ADAM10-shRNA alleviates the DOX-induced cardiac dysfunction

N-cadherin mediates adhesion in IDs at the termini of cardiomyocytes, which forms a critical mechanical junction and guides cardiomyocyte organization. Since ADAM10 is crucial for the N-cadherin cleavage, the present research focused on elucidating the possible role of ADAM10 inhibition in DOX-induced cardiomyopathy.

**Figure 4:**
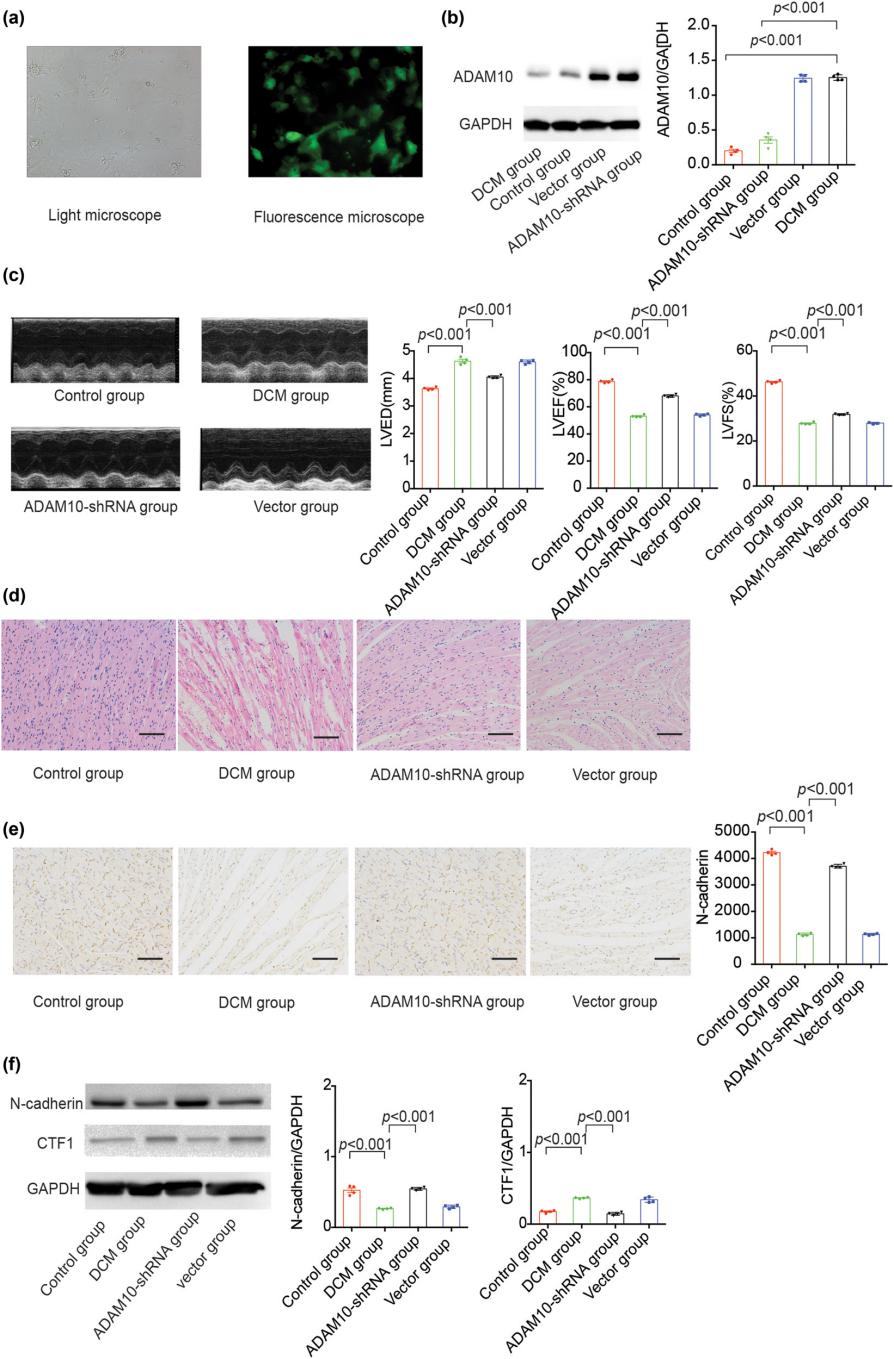
ADAM10-shRNA alleviates the DOX-induced mouse death and cardiac dysfunction. (a) Fluorescence images of primary cardiomyocytes isolated from rats in the ADAM10-shRNA group; GFP was observed under light (left) or fluorescence (right) microscopy. (b) ADAM10 expression in control and DCM groups. (c) Representative images of LV M-mode echocardiograms (left), cardiac function parameters measured by echocardiography and hemodynamic analysis (right). (d) Hematoxylin and eosin-stained myocardial samples. (e) Immunohistochemistry for N-cadherin expression in sections of cardiac tissues. (f) Western blotting analysis of N-cadherin and CTF1 levels in rats’ cardiac tissues in the control, DCM, ADAM10-shRNA, and vector groups. *P-*values in a, c, e, and f were determined using the Kruskal–Wallis test. All data are presented as the mean values ± SEM. Magnification ×200. ADAM10, disintegrin metalloproteinase 10; shRNA, short hairpin RNA; DOX, doxorubicin; GFP, green fluorescent protein; CTF1, C-terminal fragment1; DCM, dilated cardiomyopathy.

First, ADAM10-shRNA lentiviruses or blank vectors were transfected into the rats. Subsequently, the murine DCM model induced by injection of DOX was established. The fluorescence intensity in primary cardiomyocytes isolated following transfection was detected to confirm that the ADAM10-shRNA lentivirus transfection was successful. As shown in Figure 4a, GFP was observed in the cardiomyocytes from the ADAM10-shRNA group. Furthermore, to identify whether ADAM10 was altered in cardiac disease, the expression of ADAM10 was examined. Rats treated with DOX displayed higher ADAM10 expression than those in the control group. ADAM10 protein expression was markedly lower in the ADAM10-shRNA group than in the DCM group (Figure 4b). Taken together, these results demonstrated that recombinant lentivirus was successfully introduced into the rats.

Echocardiographic examination showed that DOX induced LV dilation and dysfunction, including decreased LVEF, reduced fractional shortening, and enlarged LVEDD. However, the ADAM10-shRNA lentivirus markedly attenuated the cardiac malfunction (Figure 4c). Furthermore, in H&E-stained myocardial samples, DOX treatment resulted in myofilament disarray and endocardium hyperplasia (Figure 4d). Conversely, ADAM10-shRNA suppressed these myocardial histological changes (Figure 4d). Collectively, these results demonstrated that ADAM10 inhibition could attenuate cardiac remodeling.

To further evaluate the effect of ADAM10-shRNA lentivirus on the shedding of N-cadherin in the myocardium, N-cadherin IHC assays were performed. As shown in Figure 4e, the expression of N-cadherin was significantly decreased in the IDs of myocardium from the DOX-treated rats, whereas injection of ADAM10-shRNA lentivirus resulted in enhanced N-cadherin accumulation. In line with these results, western blotting analysis revealed that DOX treatment repressed N-cadherin expression and upregulated CTF1 protein level (Figure 4f) and that these effects could be counteracted by ADAM10-shRNA lentivirus transfection. These findings indicated that the protective effect of ADAM10-shRNA on DOX-induced cardiomyopathy is associated with N-cadherin accumulation.

## 4 Discussion

Structural cardiac remodeling involves the reorganization of the heart size and shape, which affects the progression of heart failure [16]. To investigate potential therapeutic targets that could reverse heart failure progression, novel biomarkers and mechanisms involved in cardiac remodeling must be studied.

The present investigation indicated that the metalloproteinase ADAM10 plays a role in the N-cadherin ectodomain cleavage in cardiomyocytes, which results in the generation of CTF1 and therefore, regulating cell–cell adhesion. The present study further demonstrated that this cleavage is involved in the DOX-induced myocardial toxicity and cardiomyocyte contractile dysfunction. Briefly, the results collectively suggested that ADAM10 inhibition may be a potential therapeutic target for DOX-induced cardiac remodeling.

The metalloproteinase ADAM10 sheds a multitude of different membrane-bound proteins [21]. It was reported that ADAM10 is the major proteinase responsible for N-cadherin ectodomain cleavage in fibroblasts and neurons [20]. In this research, lentiviral vectors expressing shRNA-targeting ADAM10 were constructed, which can be used to inhibit ADAM10 activity. As expected, the ectodomain release of N-cadherin was lost in ADAM10-knockdown cardiomyocytes, leading to the reduction of CTF1 level. By contrast, ADAM10 activation resulted in increased CTF1 levels. Thus, the present study indicated that ADAM10 is responsible for the ectodomain shedding of N-cadherin in cardiomyocytes.

As an adhesion molecule, N-cadherin mediates homotypic and heterotypic cell–cell interaction [22]. Increased N-cadherin expression level on the cell surface enhances cell adhesion [23]. The present study revealed that ADAM10 is involved in N-cadherin-mediated intercellular adhesion. Additionally, this interaction is closely related to the increased amounts of full-length N-cadherin on the surface of ADAM10-deficient cardiomyocytes. Since N-cadherin anchors to a neighboring myocyte through its extracellular domain and is essential for cell–cell communication of cardiomyocytes *in vivo* and *in vitro* [*9*,*24*], ADAM10-dependent N-cadherin cleavage likely plays a vital role in non-ischemic cardiac diseases that are affected by the missing initial ectodomain cleavage, such as DOX-induced DCM.

The present study revealed decreased left ventricular systolic function and enlarged LVEDD in rats following DOX injection, consistent with results from previous studies [20]. The heart function depends on the coupled cardiomyocytes to form a co-functional syncytium, and the AJs act as the mechanical link between myofibrils and the ID membrane [25]. The AJ’s protein complex is composed of N-cadherin and cytosolic catenin. Furthermore, N-cadherin-deficient mice show disruption of cardiac cytoarchitectural organization, DCM, and heart failure [26]. The present study showed a close association between cardiac remodeling and loss of full-length N-cadherin in DOX-treated rats. Although a link between impaired N-cadherin and DCM has been previously described [23], the underlying mechanisms remain elusive. Considering ADAM10-dependent N-cadherin cleavage in cardiomyocytes, it is plausible to speculate that ADAM10 may be responsible for N-cadherin-loss-induced cardiac remodeling. The present study found that DCM rats exhibited ADAM10 accumulation. That is consistent with previous reports revealing that ADAM10 expression was exclusively elevated in human DCM [16], which indicated that ADAM10 may accelerate cardiac remodeling. These results demonstrated that ADAM10-shRNA increased the expression level of N-cadherin on the myocardial cell surface and rescued cardiac function in a DOX challenged murine model, suggesting that ADAM10 inhibition might serve as a novel therapeutic avenue to reverse cardiac remolding.

In summary, the current research revealed that ADAM10 is crucial for the cleavage of N-cadherin in cardiomyocytes. The ADAM10-dependent shedding of N-cadherin may regulate cardiac remodeling in DCM, which provides new insights for the management of DCM and heart failure. Inevitably, there are a few limitations to our study. As is known, ventricular remodeling in DCM is an intricate pathological process in which multiple factors may be involved. A preliminary exploration of ADAM10 was conducted in the present study, while other members of the ADAM family may also take part in the processing and hydrolysis of N-cadherin. On the other hand, there are probably other protease signaling pathways contributing to form a complex regulatory network, which will be further investigated in the future.
